# Comparison of Peak Oxygen Uptake Between Upper-Body Exercise Modes: A Systematic Literature Review and Meta-Analysis

**DOI:** 10.3389/fphys.2020.00412

**Published:** 2020-05-19

**Authors:** Julia Kathrin Baumgart, Berit Brurok, Øyvind Sandbakk

**Affiliations:** ^1^Department of Neuromedicine and Movement Science, Centre for Elite Sports Research, Norwegian University of Science and Technology, Trondheim, Norway; ^2^Department of Physical Medicine and Rehabilitation, St. Olav's University Hospital, Trondheim, Norway

**Keywords:** peak aerobic capacity, aerobic endurance, wheelchair ergometer, wheelchair treadmill, arm crank ergometer, upper-body poling

## Abstract

**Purpose:** To compare peak oxygen uptake (VO_2peak_) between the asynchronous arm crank ergometry (ACE), and synchronous wheelchair ergometry (WERG), wheelchair treadmill (WTR), and upper-body poling (UBP) mode.

**Methods:** PubMed, Scopus, CINAHL, and SPORTDiscus™ were systematically searched, and identified studies screened based on title, abstract, and thereafter full-text. Studies comparing VO_2peak_ between ≥2 of the modes were included. A meta-analysis was performed by pooling the differences in VO_2peak_ between upper-body exercise modes. The quality of the included studies was assessed and the level of evidence (LoE) established for each mode comparison. Meta-regression analyses investigated the effect of total body mass and participant-related characteristics (% of able-bodied participants, % of participants with tetraplegia and % of participants who are wheelchair athletes) on differences in VO_2peak_ between modes.

**Results:** Of the 19 studies included in this review, 14 studies investigated the difference in absolute and body-mass normalized VO_2peak_ between ACE and WERG, and 5 studies examined the differences between ACE and WTR. No significant difference in absolute or body-mass normalized VO_2peak_ was found between ACE and WERG (overall effect ±95% CI: 0.01 ± 0.06 L·min^−1^ and 0.06 ± 1.2 ml·kg^−1^·min^−1^, both *p* > 0.75; LoE: strong). No significant difference in absolute or body-mass normalized VO_2peak_ was found between ACE and WTR (overall effect ±95% CI: −0.10 ± 0.18 L·min^−1^ and −1.8 ± 2.5 ml·kg^−1^·min^−1^, both *p* > 0.14; LoE: moderate). Absolute and/or body-mass normalized VO_2peak_ did not differ between WERG and WTR in one study with 13 participants (LoE: limited) and between ACE and UBP in one study with 18 participants (LoE: moderate). In the meta-regression analyses, there was no significant effect of the investigated factors on differences in VO_2peak_.

**Conclusions:** The differences between the asynchronous ACE and synchronous WERG propulsion, including possible differences in trunk involvement, do not seem to influence VO_2peak_. Therefore, ACE and WERG can be used interchangeably to test VO_2peak_. Possible differences in VO_2peak_ in all other mode comparisons remain unclear due to the wide CIs and limited to moderate LoE.

## Introduction

In individuals who primarily use their upper-body during exercise, peak oxygen uptake (VO_2peak_) attained during maximal effort exercise is commonly used as an indicator of cardiorespiratory fitness. VO_2peak_ is dependent on genetic predisposal, the level of physical activity, and type of disability of the individual tested. Particularly low VO_2peak_ values have been found among non-active individuals with a spinal cord injury (SCI) with high lesion levels and a correspondingly reduced muscle mass (Janssen et al., 2002; de Groot et al., 2016). In comparison, especially Paralympic (Para) athletes who compete in various sitting endurance sports, display relatively high VO_2peak_ (Bernardi et al., 2012; Baumgart et al., 2018a). Arm crank ergometry (ACE) is the most commonly used test mode to assess upper-body VO_2peak_. However, in a sports-context, the specificity of the test mode is of importance to reflect a VO_2peak_ that is of relevance for the respective sport (McCafferty and Horvath, 1977). In wheelchair athletes, the wheelchair ergometry (WERG) (i.e., employing a wheelchair on rollers), or the wheelchair treadmill (WTR) mode may provide a more sport-specific alternative compared to the ACE mode. In Paralympic ice hockey players, sitting Para cross-country skiers and sitting Para biathletes, the upper-body poling (UBP) mode may be more sports-specific compared to the ACE mode for assessing VO_2peak_.

The ACE mode is more efficient than the WERG, WTR, and UBP modes partly due to the continuous rather than discontinuous power production. At the same time, during WERG, WTR, and UBP, the synchronous movement of the arms allows more displacement of the trunk compared to during ACE, where the asynchronous movement of the arms limits trunk displacement. Consequently, a higher VO_2peak_ might be expected in WERG, WTR, and UBP compared to the ACE mode, speculatively due to recruitment of more active muscle mass during the synchronous movement. Some studies have shown higher values in the WERG or WTR compared to the ACE mode (Wicks et al., 1983; Gass et al., 1995; Bloemen et al., 2015), whereas others show no differences between the WERG, WTR, and UBP and the ACE mode (Gayle et al., 1990; Martel et al., 1991; Arabi et al., 1997; Baumgart et al., 2018b). Furthermore, in a previous study, we conducted a pooled regression analysis based on 22 studies in 169 wheelchair athletes, in which the WERG/WTR mode resulted in 5 mL·kg^−1^·min^−1^ higher VO_2peak_ compared to ACE (Baumgart et al., 2018a). The higher VO_2peak_ in WERG/WTR compared to ACE in the latter analysis might be explained by inclusion of only wheelchair athletes for whom the WERG/WTR mode is more sports-specific than the ACE mode. However, the results of the review of Baumgart et al. (2018a) are based on regression analyses and need to be interpreted with caution since VO_2peak_ was not compared directly between modes in a repeated-measures design within the same studies. In this context, where there is large heterogeneity in the participants tested, a meta-analysis based solely on studies comparing VO_2peak_ between different modes in a repeated-measures design is a more valid approach due to a reduced effect of between-participant variance on the overall outcome. Furthermore, meta-regression analyses can provide information on whether wheelchair athletes achieve a higher VO_2peak_ in modes using wheelchair propulsion as compared to the ACE mode.

In addition to being specifically trained for a certain test mode, other participant-related factors might explain why there is a higher VO_2peak_ in the WERG and WTR compared to the ACE mode in some studies, while there are no differences between modes in other studies. Speculatively, persons with a complete tetraplegia, who have reduced sitting balance, might be able to exhaust themselves more in modes with less displacement in the upper-body (i.e., the ACE), thereby reducing the differences in VO_2peak_ between the ACE and the other modes. In addition, differences between the ACE and the other modes might be influenced by the % of able-bodied participants often included in these types of studies, since able-bodied participants are generally less familiar to using a wheelchair compared to wheelchair users. Furthermore, the influence of total body mass on the difference in VO_2peak_ between upper-body exercise modes has not yet been investigated. Overall, investigating these factors would contribute to the understanding of the variability in VO_2peak_ differences between modes across studies.

Information on whether VO_2peak_ differs between upper-body test modes provides important knowledge both in a clinical as well as in a sport setting and indicates to what extent test modes can be used interchangeably. Therefore, the aim of this systematic literature review and meta-analysis was to compare VO_2peak_ between the ACE, WERG, WTR, and UBP. Furthermore, the influence of other participant characteristics (i.e., body mass, % of able-bodied participants, % of participants with tetraplegia, and % of participants who are wheelchair athletes) was investigated in meta-regression analyses. It is hypothesized that VO_2peak_ is higher in WERG, WTR, and UBP compared to ACE. In addition, it is hypothesized that VO_2peak_ is higher in the ACE compared to other modes in studies with a higher % of able-bodied participants and a higher % of participants with tetraplegia. Furthermore, VO_2peak_ is expected to be higher in WERG and WTR as compared to ACE in studies with a higher % of participants who are wheelchair athletes.

## Methods

This review was conducted in accordance with the Preferred Reporting Items for Systematic Review and Meta-Analysis Protocols (PRISMA-P) guidelines [11] (see Supplementary Material S1 for the PRISMA checklist). Additionally, the study protocol was registered a priori in the International Prospective Register of Systematic Literature Reviews (PROSPERO) under the following registration number: CRD42019025063.

### Data Sources and Search Strategy

PubMed, CINAHL (through EBSCOhost), SPORTDiscus™ (through EBSCOhost) and Scopus^®^ were systematically searched in November 2018 using relevant keywords and a Boolean search string (see Supplementary Material S2 for the Boolean search string).

References of the included studies were searched manually for further identification of studies relevant to the research question.

### Inclusion Criteria

Studies were included if they compared absolute and/or body-mass normalized VO_2peak_ between at least two of the following upper-body exercise modes: ACE, WERG, WTR, and UBP. Only studies where the same participants were tested in a repeated-measures design in two or more modes in the respective study were included, i.e., studies were excluded if the participants were split in groups and tested in one of the modes only. There were no restrictions made to the participants tested, i.e., included were articles comparing VO_2peak_ between upper-body exercise modes in able-bodied participants as well as participants that range from untrained individuals who ambulate in a wheelchair for various reasons to Paralympic athletes in a variety of sports. Studies were included if they tested absolute and/or body-mass normalized VO_2peak_ in a standardized laboratory setting and the same ergospirometer was used within each study. Field studies were excluded due to a lack of standardization. Furthermore, studies that included an intervention in between VO_2peak_ testing in the two or more modes were excluded. Only full-text, original research published in peer-reviewed journals in English, Dutch, German or French were considered. Abstracts and conference proceedings were not eligible due to lack of reporting detailed methods and results. No restrictions were made on the publication date of the studies.

### Study Selection

After eliminating duplicate articles, the titles were screened by JB. Studies that did not directly mention VO_2peak_ or a synonym in the study title but were likely to have included it as a secondary measure, were also included. In a second step, the abstracts of the studies deemed relevant by title were read by JB. Articles considered relevant by abstract were then read in full-text by JB and BB. There was full agreement on the inclusion of full-text articles. JB and BB were not blinded to the names of the authors of the studies.

### Assessment of Methodological Quality

The quality of the included studies was assessed by JB and BB with a modified version of the Downs and Black checklist (Downs and Black, 1998) (see Supplementary Material S3 for the modified Downs and Black checklist). Modified versions of this checklist have been employed in several reviews in the field of sports science, which also mainly used cross-sectional studies for their data retrieval (Hebert-Losier et al., 2014; Baumgart et al., 2018a). The original checklist comprises 27 items, which are distributed over five sub-scales: reporting (item 1–10), external validity (item 11–13), bias (item 14–20), confounding (items 21–26), and power (item 27) [13]. For the purpose of this review, items 8, 9, 11–16, 19, and 22–26 were excluded since our review did not focus on interventions The term “patient” was replaced by participant and “treatment” was interpreted in the context of testing (Hebert-Losier et al., 2014; Baumgart et al., 2018a). All items, except item number 5 and 27, were rated as “Yes” (1 point), “No” (0 points), or “Unknown” (0 points). For item 5, sex, age, body mass, type of disability, physical activity level, and test protocol (i.e., increment duration) were considered to be core confounders. Item 5 was scored with 2 points if all core confounders were mentioned. 1 point was scored if 5 out of the 6 core confounders were explained. Item 27 was scored with 3 points for studies with above 21 participants, 2 points with 18–21 participants, 1 point with 15–17 participants and 0 points with 15 or fewer participants. Quality cut-off points were decided on retrospectively and studies were ranked to be of low (0–6 points), moderate (7–11 points), or good methodological quality (12–18 points) based on the score of the Downs and Black checklist. The level of evidence (LoE) for each mode comparison was ranked from unknown to strong by combining the quality scores of each of the studies included in the respective mode comparison (see Table 1).

**Table 1:** Criteria for determining the level of evidence based on the quality of the studies included for each modes comparison (adjusted from the criteria provided by van Tulder et al., 2003).

**Level**	**Criteria**
Strong	Data provided in multiple studies of good methodological quality OR in one study of good methodological quality and multiple studies of moderate methodological quality
Moderate	Data provided in multiple studies of moderate methodological quality OR in one study of good methodological quality
Limited	Data provided in one study of moderate methodological quality
Very limited	Data provided in one study of low quality

### Data Extraction

Data on VO_2peak_ in the respective mode and the characteristics of the participants (number of participants, sex, number of able-bodied participants, number of athletes who are wheelchair athletes, age, body mass, type of disability and training status) as well as the starting workload, duration and workload increases of the increments used during the test protocols was extracted from the included studies by JB with the BB cross-checking all the data.

### Statistics

All data are presented as mean ± standard deviation (SD) or 95% confidence intervals (CI) unless specified otherwise. A meta-analysis was performed in Stata 14.2 (StataCorp LLC, Texas, USA) by the random effects approach described by Dersimonian and Laird (1986) to investigate whether there were differences in VO_2peak_ between upper-body exercise modes (see Supplementary Material S4 for the input for the meta-analyses). Participants of the included studies were heterogeneous with respect to body mass, being specifically trained for a certain test mode and whether they had a disability or not; and in case they had a disability with respect to the type of disability. Therefore, random-effects meta-regression analyses with REML estimates of heterogeneity and a Knapp and Hartung modification (Knapp and Hartung, 2003) were performed to look into the separate effect of each of the following factors on VO_2peak_: body mass (kg), % of able-bodied participants, % of participants with tetraplegia, and % of participants who are wheelchair athletes (see Supplementary Material S4 for the input for the meta-regression analyses). The % of able-bodied participants ranged from 100 in studies that solely tested able-bodied participants to 0 for studies that solely tested participants with a disability. The % of participants with tetraplegia ranged from 100 in studies that solely tested participants with tetraplegia to 0 for studies that solely tested participants without tetraplegia. The % of participants who are wheelchair athletes ranged from 100 in studies that solely tested wheelchair athletes to 0 for studies that solely tested participants who are no wheelchair athletes. An alpha level of 0.05 was used to indicate statistical significance.

## Results

### Study Selection

**Figure 1:**
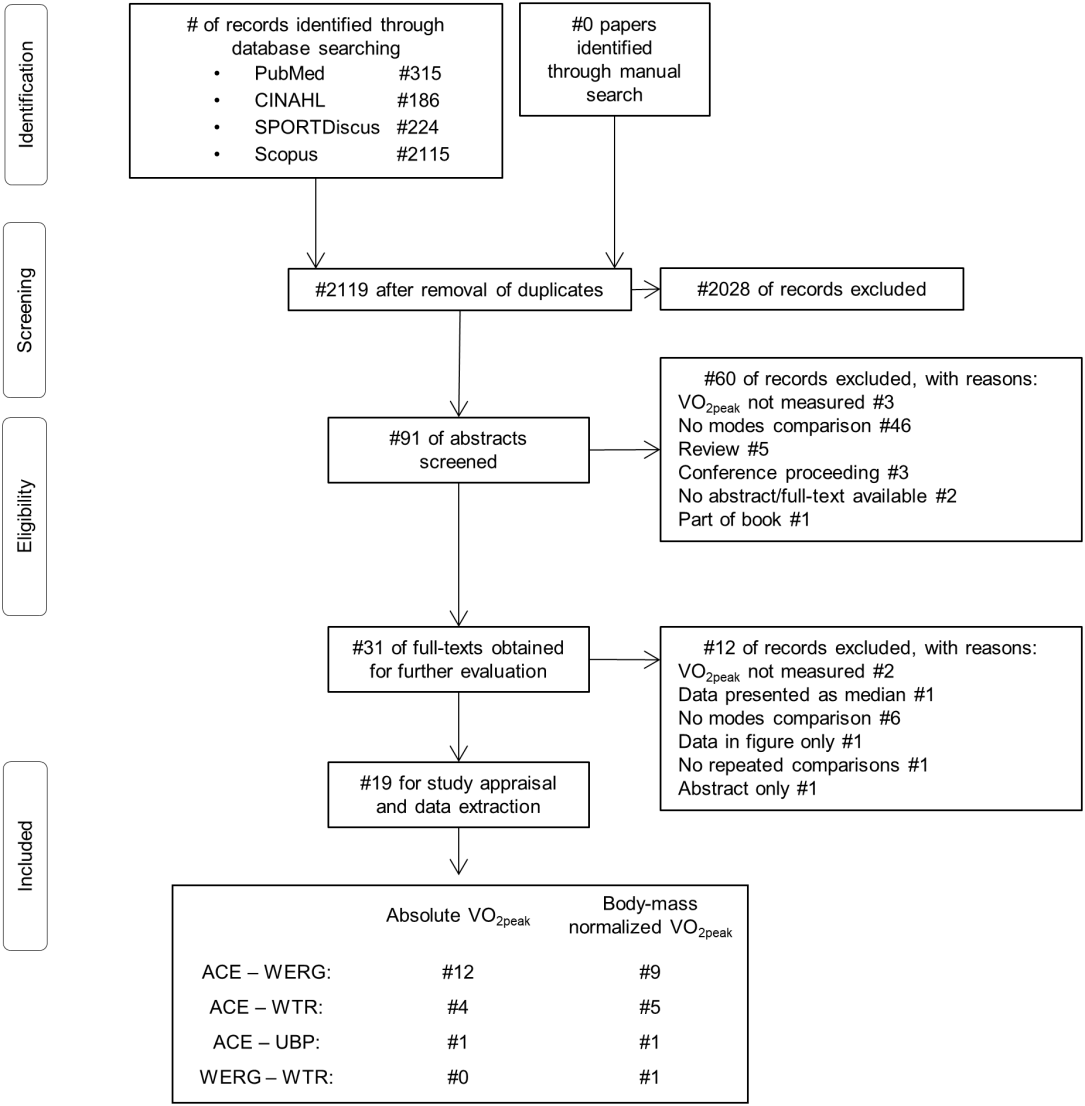
Preferred Reporting Items for Systematic Reviews and Meta-Analyses (PRISMA) flowchart depicting the study identification, screening, eligibility and inclusion process. VO_2peak_, Peak oxygen uptake; ACE, arm crank ergometry; WERG, wheelchair ergometry; WTR, wheelchair treadmill; UBP, upper-body poling.

Of the 2119 studies initially screened on their title, 19 full-text studies were included in this systematic literature review (Figure 1). Of these, 12 and nine studies compared absolute or body-mass normalized VO_2peak_ values, respectively, in 239 and 200 participants, between the ACE and the WERG mode. Four and five studies compared absolute and body-mass normalized VO_2peak_, respectively, in 43 and 51 participants, between the ACE and the WTR modes. VO_2peak_ was compared between the WERG and WTR mode in one study including 13 participants (Arabi et al., 1997) and between the ACE and UBP mode in one study including 18 participants (Baumgart et al., 2018b).

### Methodological Quality

**Figure 2:**
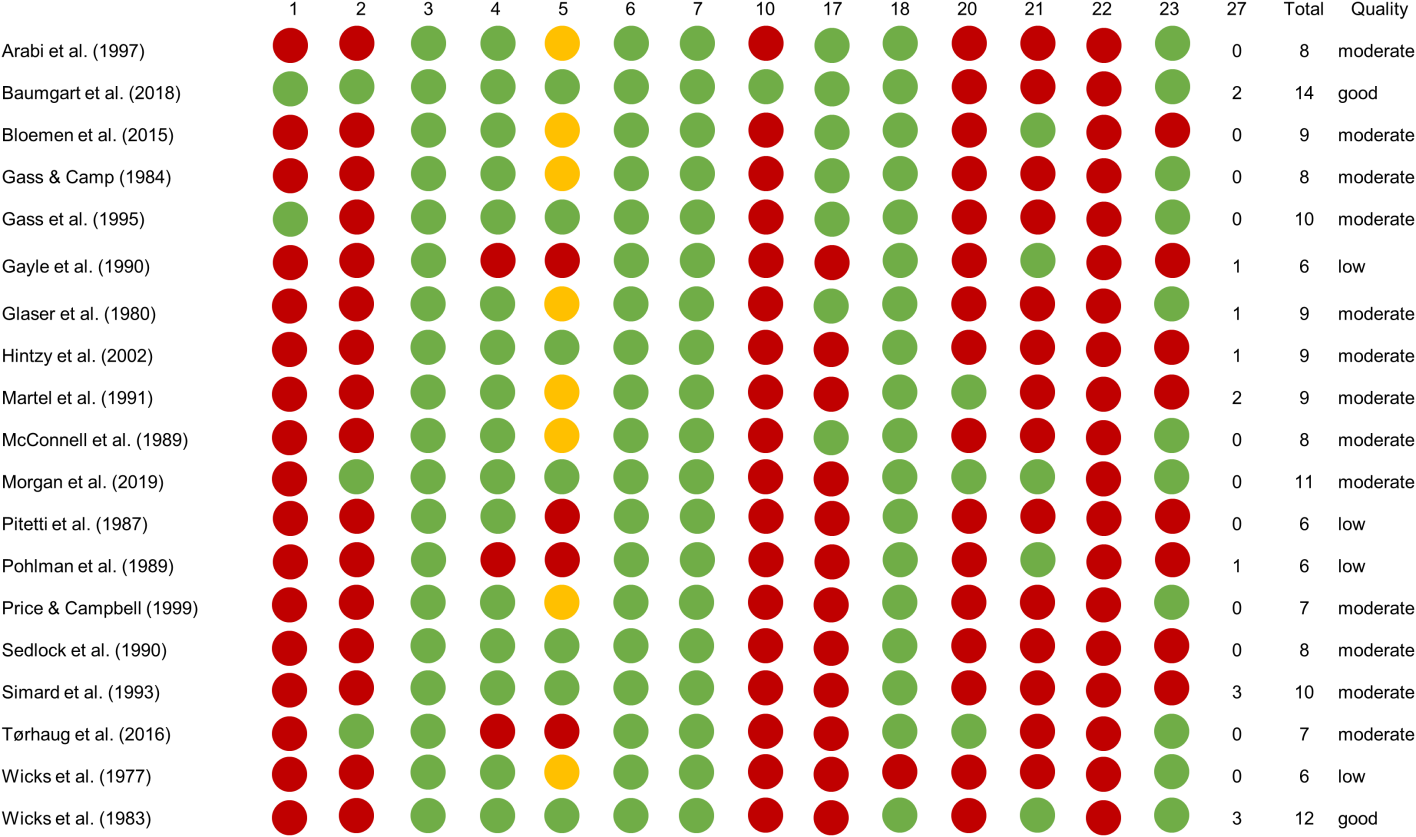
Quality scores of the 19 included studies. Green dots are for items scored Yes, red dots for items scored No and yellow dots for “Partial Yes” (i.e., scoring 1 of the 2 points possible for item 5).

There was an 84% agreement between JB and BB in the items rated initially, with full agreement reached when re-checking details in the appraised studies. Two studies were ranked as having good, 13 as having moderate, and 4 as having low methodological quality (Figure 2). The quality of the studies included in each of the mode comparisons determines the LoE of the respective comparison.

### Meta-Analyses: Comparison of VO_2peak_ Between Modes

**Figure 3:**
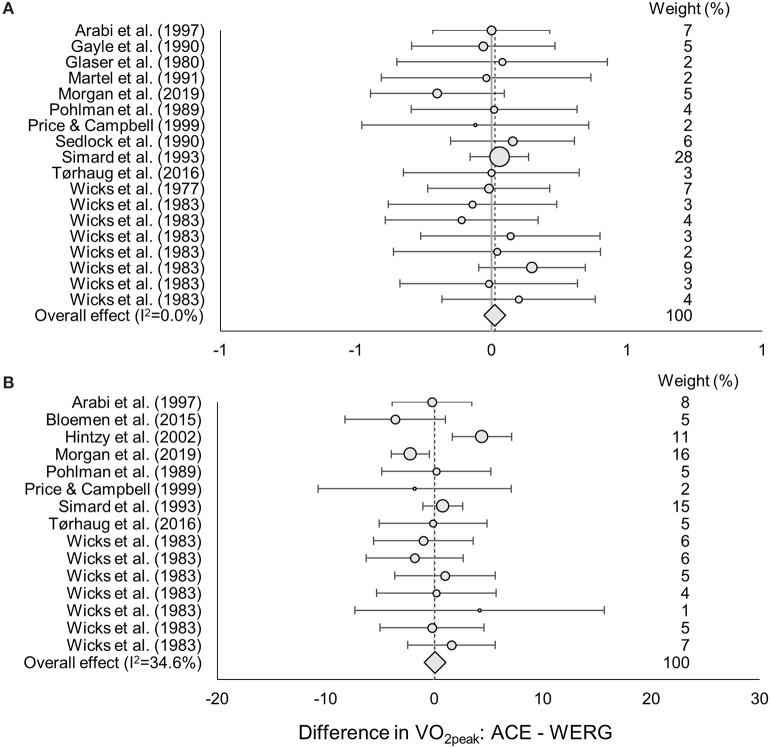
ES, Effect size (95% CI range) of the difference in **(A)** absolute and **(B)** body-mass normalized VO_2peak_ between the arm crank ergometer vs. wheelchair ergometer mode. The dot size indicates the relative weight of each study in determining the overall effect size.

**Figure 4:**
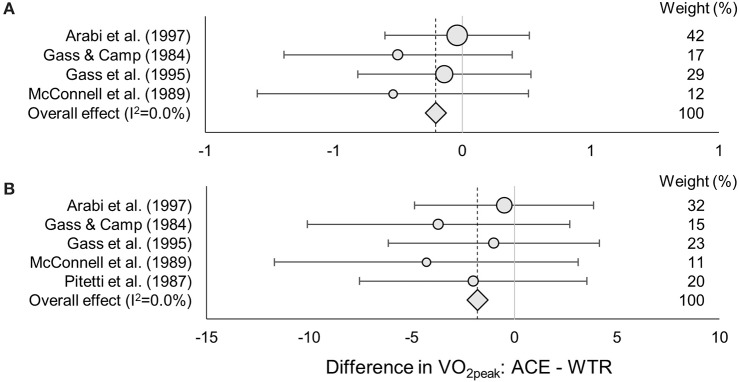
ES, Effect size (95% CI range) of the difference in absolute **(A)** and body-mass normalized **(B)** VO_2peak_ between the arm crank ergometer vs. wheelchair ergometer mode. The dot size indicates the relative weight of each study in determining the overall effect size.

No differences in absolute or body-mass normalized VO_2peak_ were found between testing in the ACE or WERG mode (overall effect ± 95% CI: 0.01 ± 0.06 L·min^−1^, *p* = 0.75, and 0.06 ± 1.2 ml·kg^−1^·min^−1^, *p* = 0.93; LoE: strong) (Figure 3). No difference in absolute or body-mass normalized VO_2peak_ were found between testing in the ACE or WTR mode (overall effect ± 95% CI: −0.10 ± 0.18 L·min^−1^, *p* = 0.28, and −1.8 ± 2.5 ml·kg^−1^·min^−1^, *p* = 0.14; LoE: moderate) (Figure 4). One study compared body-mass normalized VO_2peak_ between the WERG and the WTR mode and found no difference (Arabi et al., 1997) (LoE: limited). In addition, one study compared absolute and body-mass normalized VO_2peak_ between the ACE and the UBP mode and found no difference (Baumgart et al., 2018b) (LoE: moderate). The data extracted for each of the included studies is found in Tables 2, 3, 4.

**Table 2:** Data extraction table.

**References**	**VO**_****2peak****_ **values**						
	**Absolute VO_**2peak**_**	**Absolute VO_**2peak**_**	**BMN VO_**2peak**_**	**BMN VO_**2peak**_**	**Peak power output (W)**	**Peak power output (W)**	**Quality appraisal**
**COMPARISON ACE-WERG**	**ACE**	**WERG**	**ACE**	**WERG**	**ACE**	**WERG**	**Strong**
Arabi et al. (1997)*	1.23 ± 0.35	1.23 ± 0.19	19.0 ± 5.2	19.2 ± 4.3	78 ± 19	ns	Moderate
Bloemen et al. (2015)	ns	ns	19.5 ± 4.4	23.1 ± 7.3	ns	ns	Moderate
Gayle et al. (1990)	1.95 ± 0.35	1.98 ± 0.39	ns	ns	ns	ns	Moderate
Glaser et al. (1980)	1.77 ± 0.56	1.73 ± 0.56	ns	ns	ns	ns	Moderate
Hintzy et al. (2002)	ns	ns	38.9 ± 4.0	34.5 ± 3.6	111 ± 10	79 ± 12	Moderate
Martel et al. (1991)	1.88 ± 0.62	1.9 ± 0.63	ns	ns	97 ± 25	74 ± 19	Moderate
Morgan et al. (2019)	1.2 ± 0.25	1.4 ± 0.31	15.9 ± 2.0	18.1 ± 2.3	61 ± 13	62 ± 17	Moderate
Pohlman et al. (1989)	1.95 ± 0.46	1.94 ± 0.39	27.5 ± 6.2	27.3 ± 7.7	ns	ns	Low
Price and Campbell (1999)	1.90 ± 0.4	1.96 ± 0.4	29.7 ± 8.2	31.5 ± 8.8	125 ± 24	55 ± 31	Moderate
Sedlock et al. (1990)	1.27 ± 0.29	1.19 ± 0.19	ns	ns	63 ± 4	35 ± 2	Moderate
Simard et al. (1993)	0.83 ± 0.28	0.80 ± 0.27	13.0 ± 4.6	12.2 ± 4.7	23 ± 16	14 ± 17	Moderate
Tørhaug et al. (2016)	2.2 ± 0.4	2.2 ± 0.4	27.3 ± 5.7	27.4 ± 6.7	130	100	Moderate
Wicks et al. (1977)	1.41 ± 0.21	1.42 ± 0.22	ns	ns	ns	ns	Low
Wicks et al. (1983)	0.93 ± 0.28	1 ± 0.35	13.7 ± 4.1	14.7 ± 5.2	34 ± 8	8 ± 3	Good
	0.83 ± 0.14	0.94 ± 0.29	13.1 ± 2.2	14.9 ± 4.6	43 ± 11	8 ± 3	
	1.62 ± 0.42	1.55 ± 0.37	23 ± 5.9	22 ± 5.2	89 ± 26	34 ± 8	
	1.97 ± 0.42	1.95 ± 0.45	28 ± 6.2	27.8 ± 6.4	113 ± 16	41 ± 7	
	1.23 ± 0.12	1.08 ± 0.16	21.7 ± 8.3	17.5 ± 8.3	65 ± 16	28 ± 5	
	2.01 ± 0.43	2.02 ± 0.54	31 ± 6.6	31.2 ± 7.6	116 ± 19	42 ± 7	
	2.26 ± 0.36	2.16 ± 0.28	37.7 ± 4.5	36.1 ± 4.7	121 ± 9	40 ± 7	
**COMPARISON ACE-WTR**	**ACE**	**WTR**	**ACE**	**WTR**	**ACE**	**WTR**	**Moderate**
Arabi et al. (1997)*	1.23 ± 0.35	1.25 ± 0.38	19 ± 5.2	19.5 ± 6.1	78 ± 19	ns	Moderate
Gass and Camp (1984)	1.96 ± 0.47	2.21 ± 0.54	30.1 ± 6.6	33.8 ± 7.9	ns	ns	Moderate
Gass et al. (1995)	1.65 ± 0.42	1.72 ± 0.30	23.8 ± 6.0	24.8 ± 5.1	ns	ns	Moderate
McConnell et al. (1989)	2.15 ± 0.58	2.42 ± 0.68	30.3 ± 7.7	34.6 ± 9.9	ns	ns	Moderate
Pitetti et al. (1987)	ns	ns	31.4 ± 5.7	33.4 ± 5.7	ns	ns	Low
**COMPARISON ACE-UBP**	**ACE**	**UBP**	**ACE**	**UBP**	**ACE**	**UBP**	**Moderate**
Baumgart et al. (2018b)	ns	ns	40.3 ± 7.3	39.5 ± 6.6	152 ± 29	127 ± 31	Good
	ns	ns	32.7 ± 7.0	30.3 ± 6.1	146 ± 33	118 ± 34	
**COMPARISON WTR-WERG**	**WTR**	**WERG**	**WTR**	**WERG**	**WTR**	**WERG**	**Limited**
Arabi et al. (1997)*	1.25 ± 0.38	1.23 ± 0.19	19.5 ± 6.1	19.2 ± 4.3	ns	ns	Moderate

*Absolute (L·min^−1^) and body-mass normalized (BMN) (ml·kg^−1^·min^−1^) VO_2peak_ values, peak power output (Watt) as well as information on the methodological quality for each of the studies included per mode comparison. The level of evidence for each mode comparison, which is based on the quality of the included studies, is provided in the last column in the row highlighted in gray. Data are presented as means ± SD*.

*VO_2peak_, Peak oxygen uptake; BMN, body-mass normalized; ACE, arm crank ergometry; WERG, wheelchair ergometry; WTR, wheelchair treadmill; UBP, upper-body poling; ns, not specified*.

**Table 3:** Data extraction table (continued).

	**Total # of participants**	**# of men/boys**	**# of women/girls**	**# of able-bodied participants**	**# of participants who are wheelchair athletes**	**Age (years)**	**Body mass (kg)**	**Type of disability**	**Physical activity level**
**COMPARISON ACE-WERG**									
Arabi et al. (1997)^*^	13	11	2	0	0	29.8 ± 8.7	ns	11 PARA, 1 AMP, 1 PM	13 NPA
Bloemen et al. (2015)	13^#^	9	4	0	0	13.4 ± 3.5	46.2 ± 18.7	13 SB	ns
Gayle et al. (1990)	15	15	0	0	3	27.0 ± 5.5	73.4 ± 14.3	15 PARA	12 PA, 3 A
Glaser et al. (1980)	16	11	5	10	0	25.3 ± 3.7	66.9 ± 12.6	10 AB, 3 SB, 2 SCI, 1 PM	ns
Hintzy et al. (2002)	15	15	0	15	0	23 ± 2	74.1 ± 6.3	15 AB	NPA
Martel et al. (1991)	20	20	0	0	20	26.8 ± 7.2	ns	20 PARA	12 PA, 8 A
Morgan et al. (2019)	10	10	0	0	0	33 ± 19.6	75.7 ± 11.6	7 TETRA, 3 PARA/SB	7 PA, 3 NPA
Pohlman et al. (1989)	15	15	0	0	0	27 ± 5.5	73.4 ± 14.3	15 PARA	ns
Price and Campbell (1999)	7	ns	ns	0	7	29.3 ± 5.9	64.3 ± 1.7	7 PARA	7 A
Sedlock et al. (1990)	9	0	9	9	0	24 ± 2.3	58.6 ± 9.5	9 AB	9 NPA
Simard et al. (1993)	50	41	9	0	ns	34.1 ± 9.5	66.4 ± 12	50 TETRA	ns
Tørhaug et al. (2016)	12	12	0	0	0	47 ± 9.4	82.2 ± 16.8	12 PARA	12 NPA
Wicks et al. (1977)	7	4	3	2	2	28.1 ± 4.1	ns	2 AB, 2 TETRA, 3 PARA	7 NPA
Wicks et al. (1983)	8	8	0	0	8	28.6 ± 6	67.9 ± 14.7	8 TETRA	65 A
	5	5	0	0	5	28.8 ± 4	63.3 ± 9.7	5 TETRA	
	11	11	0	0	11	30.2 ± 8	70.5 ± 13.6	11 PARA/SB	
	10	10	0	0	10	27.5 ± 7.4	70.3 ± 16.9	10 PARA/SB	
	4	0	4	0	4	28.2 ± 7.9	61.5 ± 16.9	4 PARA/SB	
	17	17	0	0	17	26.1 ± 6.5	64.8 ± 14.1	17 PARA/SB	
	10	10	0	0	10	36 ± 4.7	59.9 ± 7.5	10 PARA/SB	
**COMPARISON ACE-WTR**									
Arabi et al. (1997)^*^	13	11	2	0	0	29.8 ± 8.7	ns	11 PARA, 1 AMP, 1 PM	13 NPA
Gass and Camp (1984)	10	10	0	0	ns	30 ± 3.2	65.3 ± 10.6^‡^	8 PARA, 1TM, 1 PM	10 PA
Gass et al. (1995)	9	9	0	0	0	30.8 ± 2.4	70.2 ± 10.1^‡^	8 PARA, 1 TM	5 NPA, 4 PA
McConnell et al. (1989)	11	11	0	0	0	26.0 ± 4.5	70.7 ± 8.6	11 PARA	ns
Pitetti et al. (1987)	8	8	0	0	0	29 ± 2.8	ns	7 PARA, 1 AMP	8 PA
**COMPARISON ACE-UBP**									
Baumgart et al. (2018b)	11	9	2	11	0	22.4 ± 2.6	78.1 ± 6.2	9 AB	11 A
	7	6	1	0	0	33.8 ± 11.2	74.4 ± 12.5	5 PARA, 1 SB	3 PA, 4 A
**COMPARISON WTR-WERG**									
Arabi et al. (1997)^*^	13	11	2	0	0	29.8 ± 8.7	ns	11 PARA, 1 AMP, 1 PM	13 NPA

*Information on the number of participants, sex, number of able-bodied participants, number of participants who are wheelchair athletes, age (years), body mass (kg), type of disability, physical activity level for each of the studies included per mode comparison. Where applicable, data are presented as means ± SD*.

*ACE, Arm crank ergometry; WERG, wheelchair ergometry; WTR, wheelchair treadmill; UBP, upper-body poling; participants with: PARA, paraplegia; TETRA, tetraplegia; SCI, participants with a spinal cord injury without specified level of the injury; AMP, participants with amputation; SB, participants with spina bifida; PM, participants with poliomyelitis; TM, participants with transverse myelitis; participants who are: A, athletes; PA, physically active; NPA, not physically active; ns, not specified*.

* *Study included for more than one modes comparison*.

‡ *Mean_pooled ± SD_pooled of the body mass at two test instances*.

# *only 11 children were able to complete the test*.

**Table 4:** Data extraction table (continued).

**References**	**Test protocol**	**Starting load**	**Starting load**	**Increments**	**Increments**
**COMPARISON ACE-WERG**		**ACE**	**WERG**	**ACE**	**WERG**
Arabi et al. (1997)^*^	Continuous	10 W	1 km/h	10 W/2 min	1 km/2 min
Bloemen et al. (2015)	Continuous	10 W	60–120 rpm	10 W/2 min	0.1 torque/min
Gayle et al. (1990)	Discontinuous	ns	ns	ns	1 km/h/min
Glaser et al. (1980)	Discontinuous	PO at 75% HR_peak_	PO at 75% HR_peak_	60 kpm (9.8 W)/min	60 kpm/min
Hintzy et al. (2002)	Continuous	20 W	20 W	10 W/2 min	10 W/2 min
Martel et al. (1991)	Discontinuous	5 W	5 W	10 W/min	10 W/min
Morgan et al. (2019)	Continuous	10 W	10 W	7 W/min	7 W/min
Pohlman et al. (1989)	Continuous	ns	ns	8.5 W/min	1 km/h/min
Price and Campbell (1999)	Continuous	30 W	30 W	5 W/min	5 W/min
Sedlock et al. (1990)	Discontinuous	12.5 W	12.5 W	12.5 W/6 min	6 W/6 min
Simard et al. (1993)	Continuous	0 W	0 W	10 W/2 min	10 W/2 min
Tørhaug et al. (2016)	Continuous	ns	ns	5–15 W/min	5–15 W/min
Wicks et al. (1977)	Continuous	60 rpm	20 rpm	100 kpm (16.3 W)/min for PARA or 25–50 kpm (8.2 W)/min for TETRA	10 rpm/min increments for PARA or 5 rpm/min increments for TETRA
Wicks et al. (1983)	Continuous	60 rpm	20 rpm	100 kpm (16.3 W)/min for PARA or 50 kpm (8.2 W)/min for TETRA	10 rpm/min increments for PARA or 5 rpm/min increments for TETRA
**COMPARISON ACE-WTR**		**ACE**	**WTR**	**ACE**	**WTR**
Arabi et al. (1997)^*^	Continuous	10 W	2 km/h, 1.5%	10 W/2 min	1 km/h/2 min
Gass and Camp (1984)	Continuous	30 W	5 km/h, 0%	5 W/20 s	0.5 km/min. +2% at 2 and 6 min
Gass et al. (1995)	Continuous	20 W	3.5 km/h, 0%	5 W/30 s	0.5 km/h or 0.5% /30 s until 4%, then 0.5 km/h /30 s
McConnell et al. (1989)	Continuous	0 kpm (0 W), 72 rpm	3.2 km/h, 0%	1 kpm/3 min	2%/3 min
Pitetti et al. (1987)	Continuous	3.2 km/h^‡^, 0%	75 kpm (12.3 W), 50 rpm	0.8–1.6 km/h increase until 7.2 km/h^‡^ then 1%/min	75 kpm (12.3 W)/min
**COMPARISON ACE-UBP**		**ACE**	**UBP**	**ACE**	**UBP**
Baumgart et al. (2018b)	Continuous	PO of RPE 11	PO at RPE 11	10 W/min	10 W/min
**COMPARISON WTR-WERG**		**WTR**	**WERG**	**WTR**	**WERG**
Arabi et al. (1997)^*^	Continuous	10 W	1 km/h	10 W/2 min	1 km/2 min

*Type of test protocol (continuous/discontinuous), starting load and increments [speed in kilometer per hour (km/h), incline in percent (%), power output in kilopond-meter (kpm) or watt (W), revolutions per minute (rpm)] for each of the studies included per mode comparison*.

* *Study included for more than one modes comparison*.

‡ *Recalculated speed in kilometers per hour (km/h) instead of miles per hour (mph)*.

*ACE, Arm crank ergometry; WERG, wheelchair ergometry; WTR, wheelchair treadmill; UBP, upper-body poling; participants with: PARA, paraplegia; TETRA, tetraplegia; PO, power output; HR_peak_, peak heart rate; RPE, rate of perceived exertion; ns, not specified*.

### Meta-Regression Analyses: Influence of Participant and Test Characteristics on VO_2peak_

We were only able to investigate the influence of body mass and participant-related characteristics on differences in VO_2peak_ between modes in the comparison of the ACE to the WERG mode due to a sufficient number of studies included for this comparison. The meta-regression analyses were based on 12 studies that provided data of 18 subgroups for the prediction of absolute VO_2peak_, and on 9 studies that provided data of 14 subgroups for the prediction of body-mass normalized VO_2peak_ (Tables 2, 3). Note that there are more subgroups than studies, since Wicks et al. (1983) presented their data in seven sub-groups. None of the investigated factors significantly predicted absolute or body-mass normalized VO_2peak_ differences between the ACE and WERG mode (Table 5).

**Table 5:** Meta-regression results on the separate influence of participant-related characteristics on differences in VO_2peak_ between the arm crank ergometer (ACE) and the wheelchair ergometer mode (WERG).

	**Coefficient [95% CI]**	***p*-value**	**Constant [95% CI]**	***p*-value**	**Tau^**2**^**	**$I_{\text{res}}^{2}$ (%)**	**$R_{\text{adj}}^{2}$ (%)**	**# of subgroups**
**EFFECT SIZE—DIFFERENCES IN ABSOLUTE VO**_**2peak**_**: ACE-WERG**								
Body mass (kg)	−0.009 [−0.02–0.004]	0.16	0.6 [−0.2–1.4]	0.15	0	0	0	15
% of able-bodied participants	0.0006 [−0.002–0.003]	0.60	0.007 [−0.06–0.07]	0.83	0	0	0	18
% of participants with a tetraplegia	−0.0005 [−0.002–0.001]	0.41	0.04 [−0.05–0.1]	0.39	0	0	0	18
% of participants who are wheelchair athletes	0.0005 [−0.001–0.002]	0.49	−0.02 [−0.1–0.1]	0.69	0	0	0	17
**EFFECT SIZE—DIFFERENCES IN BODY-MASS NORMALIZED VO**_**2peak**_**: ACE-WERG**								
Body mass (kg)	0.07 [−0.1–0.3]	0.43	−5.0 [−18.6–8.6]	0.44	2.9	44	−11	14
% of able-bodied participants	–	–	–	–	–	–	–	–
% of participants with a tetraplegia	−0.01[−0.05−0.02]	0.34	0.6 [−1.2−2.4]	0.50	2.2	32	2	15
% of participants who are wheelchair athletes	0.002 [−0.03−0.04]	0.91	−0.1 [−2.4 −2.2]	0.90	3.1	40	−13	14

*Note that there are no coefficients for the influence of % of able-bodied participants on difference in body-mass normalized VO_2peak_ between the ACE and the WERG mode, since only one study with able-bodied participants was included for this comparison*.

## Discussion

The main aim of this systematic literature review and meta-analysis was to compare VO_2peak_ between the ACE, WERG, WTR, and UBP modes in a variety participants. In brief, no difference in absolute or body-mass normalized VO_2peak_ was found either between the ACE and WERG (LoE: strong) or between the ACE and WTR mode (LoE: moderate), while the single studies comparing VO_2peak_ between the WERG and WTR mode (LoE: limited) and between the ACE and the UBP mode (LoE: moderate) found no significant differences. In the meta-regression analyses, none of the investigated factors significantly predicted differences between absolute or body-mass normalized VO_2peak_.

In the current meta-analysis, we found no difference in VO_2peak_ between the ACE and WERG mode, with a strong LoE. This is contrary to our hypothesis and indicates that more displacement of the trunk in the WERG mode does not necessarily lead to a higher active muscle mass and a consequently higher VO_2peak_. The finding is also in contrast to a previous systematic literature review, showing that the WERG/WTR mode resulted in 5 mL·kg^−1^·min^−1^ higher VO_2peak_ values compared to ACE in wheelchair athletes (Baumgart et al., 2018a). However, the higher VO_2peak_ in WERG/WTR compared to ACE in our previous review (Baumgart et al., 2018a) might due to inclusion of only wheelchair athletes, for whom the WERG/WTR mode is more sports-specific than the ACE mode. The latter is in line with a review on able-bodied runners and cyclists where sport specificity of the test mode was suggested to be important for achieving VO_2max_ (Millet et al., 2009), as well as a study on kayakers that showed higher VO_2peak_ values in the kayak than the arm crank ergometer mode (Forbes and Chilibeck, 2007). The results in the latter review are based on regression analyses and need to be interpreted with caution though since VO_2peak_ in the different modes was not directly compared within the included studies as done in the current analyses. The contrasting finding in the current review, with no difference in VO_2peak_ between ACE and WERG, suggests less effect of sport-specificity of the test mode on VO_2peak_ since also non-athlete participants with a disability and able-bodied participants are included. However, the meta-regression analyses revealed that also in studies with a higher % of participants who are wheelchair athletes, there was no difference in VO_2peak_ between the WERG and ACE mode. This indicates that both during the supposedly more sport-specific WERG and the less sport-specific ACE a sufficient amount of active muscle mass is recruited to elicit VO_2peak_. The results of the meta-regression analyses need to be interpreted with caution though, since only few studies were included in each prediction model. Overall, these data indicate that the ACE and WERG modes might be used interchangeably to test VO_2peak_ in persons that are not specifically trained for either of these two modes and possibly also in athletes that are specifically trained for the WERG mode.

Even though the included studies show a small but consistently lower mean VO_2peak_ in ACE compared to WTR, there was no significant overall effect of the test mode on VO_2peak_. This is contrary to our hypothesis and might suggest that trunk oscillations and shifts in center of gravity, that contribute more to propulsion in the WTR compared to the ACE mode (Vanlandewijck et al., 2001), do not necessarily lead to a larger active muscle mass with a consequently higher VO_2peak_. In line with the other mode comparisons in the current review, Arabi et al. (1997) found no difference in VO_2peak_ between WERG and WTR and Baumgart et al. (2018b) found no difference in VO_2peak_ between ACE and UBP or between WERG and WTR. However, our ability to conclude with certainty is limited due to the wide CIs of the overall effect, the limited to moderate LoE and the limited amount of studies included for these comparisons.

The meta-regression analyses showed that none of the participant-related characteristics (i.e., body mass, % of able-bodied participants, % of participants with tetraplegia and % of participants that are wheelchair athletes) influenced the difference in VO_2peak_ between the ACE and the WERG modes. This is in contrast to our hypotheses of VO_2peak_ being higher in the ACE compared to other modes in studies with participants with higher body mass, studies with a higher % of participants without disability and studies with a higher % of participants with tetraplegia; and VO_2peak_ being higher in the WERG/WTR compared to the ACE mode in studies with a higher % of participants who are wheelchair athletes. However, as already stated in the above, the results of the meta-regression analyses need to be interpreted with caution since only few studies were included in each prediction model. Furthermore, it remains to be investigated to what extent other participant-related and test protocol factors explain the higher VO_2peak_ in the WERG compared to the ACE mode in some studies, whereas there are no differences in other studies.

## Conclusion

No difference in VO_2peak_ between the ACE and WERG mode were found in the present meta-analyses, indicating that ACE and WERG may be used interchangeably to test VO_2peak_ in persons that are not specifically trained for a certain mode and possibly also in athletes that are specifically trained for the WERG mode. In addition, it remains unclear whether VO_2peak_ differs between ACE and WTR due to the wide CIs, moderate LoE and the limited amount of studies comparing VO_2peak_ between these two modes. Furthermore, we are not able to conclude on the comparison of VO_2peak_ between WERG and WTR, and ACE and UBP since only on study was included for each comparison.

## Data Availability Statement

The dataset for this study can be found in the Supplementary Material (S4 Excel file. Input_meta-analyses).

## Author Contributions

JB, BB, and ØS contributed to the conceptualization. JB and BB contributed to the methodology, the formal analysis and the investigation. JB contributed to the preparation of the writing for the original draft preparation. BB and ØS contributed to the review and editing of the writing. JB and ØS contributed to the funding acquisition.

## Conflict of Interest

The authors declare that the research was conducted in the absence of any commercial or financial relationships that could be construed as a potential conflict of interest.
