# Supplementary material for: Comparison of Peak Oxygen Uptake Between Upper-Body Exercise Modes: A Systematic Literature Review and Meta-Analysis
Source: Front Physiol. 2020 May 19;11:412. doi: 10.3389/fphys.2020.00412 (PMC7248246; doi:10.3389/fphys.2020.00412)
Supplement: Supplementary file 2 [file Table_2.pdf]

S 2. Figure. Boolean Search String

((arm AND crank*) AND ((wheelchair AND ergomet*) OR (wheelchair AND treadmill*) OR (wheelchair AND propulsion) OR ((hand AND cycling) OR (hand AND (biking OR bike)) OR handbik* OR handcycl* OR hand-cycl* OR hand-bik*) OR poling)) OR ((wheelchair AND ergomet*) AND ((wheelchair AND treadmill*) OR (wheelchair AND propulsion) OR ((hand AND cycling) OR (hand AND (biking OR bike)) OR handbik* OR handcycl* OR hand-cycl* OR hand-bik*) OR poling)) OR ((wheelchair AND treadmill*) AND ((wheelchair AND propulsion) OR ((hand AND cycling) OR (hand AND (biking OR bike)) OR handbik* OR handcycl* OR hand-cycl* OR hand-bik*) OR poling)) OR (((hand AND cycling) OR (hand AND (biking OR bike)) OR handbik* OR handcycl* OR hand-cycl* OR hand-bik*) AND poling)
