# Supplementary material for: Comparison of Peak Oxygen Uptake Between Upper-Body Exercise Modes: A Systematic Literature Review and Meta-Analysis
Source: Front Physiol. 2020 May 19;11:412. doi: 10.3389/fphys.2020.00412 (PMC7248246; doi:10.3389/fphys.2020.00412)
Supplement: Supplementary file 3 [file Table_3.pdf]

S 3. Table. Modified Downs and Black checklist. (Downs & Black, 1998)

	Yes (1)	Partially (1)	No or N/A (0)		Comments
Reporting					
1. Is the hypothesis/aim/objective of the study clearly described? This item is only rated as a Yes, if both the aim/purpose <u>and</u> the hypothesis are described. In case the study design does not allow any hypothesis or the direction of the study is so novel that no prior hypothesis can be formed, and this is made clear in the introduction, the item should be rated as a Yes.					
2. Are the main outcomes to be measured clearly described in the Introduction or Methods section? How VO_{2peak} was defined should be described for this item to be rated as a Yes. If it is only mentioned that VO_{2peak} was measure but no definition is added, this item should be rated as a No.					
3. Are the characteristics of the participants included in the study clearly described? In cohort studies and trials, inclusion and/or exclusion criteria should be given. In case-control studies, a case-definition and the source for controls should be given. For studies including participants with a disability, the type of disability should be clearly described. For athletes with a spinal cord injury it has to be indicated (at least) if they are tetraplegic or paraplegic for this item to be rated with a Yes.					
4. Are the interventions of interest clearly described? The ≥2 test modes that are compared should be clearly described. This entails that both the starting load (W/speed/rpm, ect.) and the increment load and increment duration should be reported.					
5. Are the principal confounders in each group of subjects to be compared clearly described? The confounders of our study are: sex, age, type of disability, physical activity level, body mass, test protocol (type of increments). For scoring 2 points all six confounders need to be described. The criteria for describing the type of disability in enough details are the same as for item 3. For scoring 1 point five of the six main confounders need to be mentioned.	(2)	(1)	(0)		
6. Are the main findings of the study clearly described? Simple outcome data (including mean) should be reported for all test modes employed in the respective study. Furthermore, the number of participants that were tested should be reported.					
7. Does the study provide estimates of the random variability in the data for the main outcomes? In non-normally distributed data the inter-quartile range of results should be reported. In normally distributed data the standard error, standard deviation or confidence intervals should be reported. If the distribution of the data is not described, it must be assumed that the estimates used were appropriate and the question should be answered yes.					
10. Have actual probability values been reported (e.g. 0.035 rather than 0.05) for the main outcomes except where the probability values is less than 0.001?					
Internal validity – bias					
17. Is the time period the participants have between tests roughly the same? If the time between repeated tests is at least 24 hours apart but within 2 weeks, this question should be answered yes. If the time between repeated tests is not mentioned, this question should be answered no.					
18. Were the statistical tests used to assess the main outcomes appropriate? The statistical tests used must be appropriate to the data. For example non-parametric methods should be used for small sample sizes. Where little statistical analysis has been undertaken but where there is no evidence of					

<table>
<tr><td>bias, this question should be answered yes. If the distribution of the data (normal or not) is not described it must be assumed that the estimates used were appropriate and the question should be answered yes.</td><td></td><td></td><td></td><td></td><td></td></tr>
<tr><td>20. Was the VO_{2peak} test valid?
This item should be rated as a Yes, if the criteria for verification of maximal effort are explicitly stated. Verification of maximal effort should at least contain two of the following five minimum criteria: 1) respiratory exchange ratio (RER) of 1.05 or higher, 2) a concentration of lactate in blood ([La-]b) of 7 mmol/liter or greater, and 3) a subjective rating of perceived exertion (RPE) with a BORG scale score of 15 or higher, 4) no increase in VO2 despite further increases in intensity or 5) reaching a maximal heart rate within 10 beats/min of an individual's age-predicted maximum (calculated as 220 – age-10 for upper-body exercise). Alternatively, the verification of maximal effort is also considered to be achieved in case a verification test was performed. Any studies which did not report on the verification of maximal effort or which include criteria below the above described, should be rated with a No. Deviating criteria should be specifically noted in the comments box provided to the right.</td><td></td><td></td><td></td><td></td><td></td></tr>
<tr><td colspan="6">Internal validity – confounding (selection bias)</td></tr>
<tr><td>21. Were the participants in different intervention groups (trials and cohort studies) or were the cases and controls (case-control studies) recruited from the same population? For example, participants for all comparison groups should be selected from the same source. The question should be answered unable to determine for cohort and case control studies where there is no information concerning the source of participants included in the study.</td><td></td><td></td><td></td><td></td><td></td></tr>
<tr><td>22. Were study participants in different intervention groups (trials and cohort studies) or were the cases and controls (case-control studies) recruited over the same period of time?
For a study which does not specify the time period over which participants were recruited, the question should be answered as unable to determine.</td><td></td><td></td><td></td><td></td><td></td></tr>
<tr><td>23. Was the test order randomized?
Studies, which state that the test order was randomized or counter-balanced, should be answered yes.</td><td></td><td></td><td></td><td></td><td></td></tr>
<tr><td colspan="6">Power</td></tr>
<tr><td>27. Did the study have sufficient power to detect a clinically important effect where the probability value for a difference being due to chance is less than 5%?
Sample sizes have been calculated to detect differences based on an effect size of 0.65 and an alpha level of 0.05.
<table>
<tr><td></td><td>Size of smallest intervention group</td><td></td></tr>
<tr><td>A (60%)</td><td><15</td><td>0</td></tr>
<tr><td>B (70%)</td><td>15-17</td><td>1</td></tr>
<tr><td>C (80%)</td><td>18-21</td><td>2</td></tr>
<tr><td>D (90%)</td><td>>21</td><td>3</td></tr>
</table></td><td></td><td></td><td></td><td></td><td></td></tr>
</table>

Downs, S. H., & Black, N. (1998). The feasibility of creating a checklist for the assessment of the methodological quality both of randomised and non-randomised studies of health care interventions. *J Epidemiol Community Health*, *52*(6), 377-384. Retrieved from http://jech.bmj.com/content/jech/52/6/377.full.pdf
